# Applying nanopore sequencing in the etiological diagnosis of bloodstream infection

**DOI:** 10.3389/fmicb.2025.1554965

**Published:** 2025-02-13

**Authors:** Yiqun Liao, Junjie Gong, Xiaoling Wang, Puwen Chen, Qinxing Chi, Xiaohong Chen

**Affiliations:** ^1^Department of Laboratory Medicine, First Affiliated Hospital of Gannan Medical University, Ganzhou, China; ^2^The First School of Clinical Medicine, Gannan Medical University, Ganzhou, China

**Keywords:** bloodstream infection, etiological diagnosis, high-throughput sequencing, nanopore sequencing, whole genome sequencing

## Abstract

Bloodstream infection (BSI) is a systemic infectious disease that can lead to shock, disseminated intravascular coagulation, multiorgan failure, and even death. Blood culture is considered the gold standard for the etiological diagnosis of BSI; however, blood culture is time-consuming and has a low positivity rate, which has limited its utility for early and rapid clinical diagnosis. Nanopore sequencing technology (NST), a third-generation sequencing method, offers rapid detection, real-time single-molecule sequencing, and ultra-long reads. These features enable the prompt detection of pathogens and the analysis of drug-resistant genes and genomic characteristics, thereby optimizing the clinical diagnosis and treatment of BSI. In this article, we summarize the application of NST in the etiological diagnosis of BSI.

## 1 Introduction

Bloodstream infection (BSI) is a severe infectious disease characterized by the invasion of various pathogenic microorganisms into the bloodstream, their proliferation, the release of toxins and metabolic products, leading to systemic infection, toxemia, and systemic inflammatory responses. The primary pathogens causing BSI include bacteria, fungi, and viruses, which can result in bacteremia and sepsis. In severe cases, BSI can cause shock, disseminated intravascular coagulation, multiorgan failure, and even death. According to a 6-year retrospective cohort study in the United States, the estimated national weighted incidence of sepsis among hospitalized patients is 5.9%, with a mortality rate of 15.6% (Rhee et al., 2017). In the United Kingdom, sepsis is estimated to be responsible for 100,000 hospital admissions and 37,000 deaths annually (Stevenson et al., 2016). A recent retrospective cohort study in Canada reported that the incidence of persistent bacteremia in hospitalized patients with gram-negative BSI is 6.8%, with a 30-day mortality rate of 17.2% and a 90-day mortality rate of 25.5% (Ong et al., 2024). With its significant incidence and mortality rates, BSI poses a serious threat to human life and health. Therefore, it is crucial to identify a rapid and accurate method for early BSI diagnosis to enable timely infection control and improve patient survival rates.

Identifying the pathogen is central to the precision treatment of BSI. Blood culture remains the gold standard for its etiological diagnosis. However, the positivity rate of blood culture is influenced by various factors, including the timing of blood collection, blood volume, type and quantity of blood culture bottles, and culture conditions. Additionally, blood culture has a lengthy process, typically requiring 2–5 days to complete pathogen identification and antimicrobial susceptibility testing (AST) (Society of Clinical Microbiology and Infection of China International Exchange and Promotion, Association for Medical and Healthcare et al., 2022), which is not conducive to early precise clinical therapy. Researchers have been striving to develop simple, rapid, and accurate methods for etiological diagnosis by exploring novel pathogen detection approaches, such as polymerase chain reaction (PCR), DNA chip technology, matrix-assisted laser desorption/ionization time-of-flight mass spectrometry (MALDI-TOF MS), and next-generation sequencing (NGS). Each of these methods has its advantages and limitations. For instance, PCR and DNA chip technology have limited clinical application due to low sensitivity in detecting mixed bacterial infections. MALDI-TOF MS results can be influenced by factors such as the culture medium and presence of red blood cells, with accuracy depending on the comprehensiveness of known databases. Similarly, NGS has a relatively short read length, encounters challenges in analyzing complex microbial genome structures, and the NGS equipment is associated with high cost. Conversely, the rapid advancement of nanopore sequencing technology (NST) has effectively addressed many of these issues. This article focuses on the application of NST in BSI rapid etiological diagnosis and determination of antimicrobial resistance.

## 2 Evolution of sequencing technologies

Sanger et al. (1977) introduced the double-deoxy terminator method, commonly known as the Sanger method, based on the subtraction-addition sequencing technique. This method, which laid the foundation for many subsequent sequencing technologies, is referred to as the first-generation sequencing technology. However, the Sanger method has limitations, including its reliance on electrophoresis separation, which restricts scalability, parallelism, and miniaturization. These limitations hinder its ability to sequence large-scale biological genomes and make it challenging to popularize due to the high cost.

In 2005, the advent of large-scale parallel sequencing instruments, exemplified by Roche’s 454 technology, Illumina’s Solexa technology, and ABI’s SOLID technology, marked the beginning of a new era of high-throughput sequencing (Fedurco et al., 2006; Turcatti et al., 2008; Shendure et al., 2005). This development led to the inception of NGS, which can simultaneously sequence millions of DNA fragments in parallel, substantially increasing throughput and enabling the rapid sequencing of individual genomes (Grada and Weinbrecht, 2013). However, NGS has limitations, including its short read length, which lowers the alignment rate of sequencing results, particularly in highly repetitive regions where large segments often result in abnormal alignments.

To address the short read length limitation of NGS, researchers began exploring new sequencing technologies. In 1996, David Deamer of the University of California and Daniel Branton of Harvard University proposed nanopore sequencing, a technique for detecting nucleic acid molecules as they pass through membrane channels (Kasianowicz et al., 1996). Although this technology faced numerous challenges during its early development, significant progress was made. In 2004, the National Human Genome Research Institute in the United States launched the “$1,000 Genome Sequencing Research Project,” aimed at reducing the cost of human genome sequencing to less than $1,000. This initiative encouraged increased research efforts to address technical challenges, such as base detection, passage speed through nanopores, and modification of nanopores themselves. Advancements in biochemistry, biophysics, and electronics have driven the rapid development of sequencing technologies. Third-generation sequencing (TGS) has brought a qualitative leap in genome sequencing, overcoming many of the limitations of early nanopore sequencing. Prominent technologies in this category include Pacific Biosciences’ (PacBio) single-molecule real-time sequencing and Oxford Nanopore Technology (ONT).

## 3 Principles and advantages of NST

NST is a TGS method that involves non-PCR sequencing of single-molecule DNA. Double-stranded DNA is unwound by helicase into single strands, and the resulting electrical signals are collected and processed for specific base recognition as the single strands are pulled through the protein nanopore network. In 2014, Oxford Nanopore Technologies introduced the MinION sequencing platform, whose core component is protein nanopores. A nanopore, composed of multiple proteins, is embedded in a thin, high-resistance film to form a channel in the membrane. When a transmembrane voltage is applied, different DNA bases generate distinct ion flow fluctuations as they pass through the nanopore. By analyzing the peak values of these ion flow fluctuations, the corresponding bases can be identified, enabling high-speed real-time sequencing.

Compared with NGS, NST offers several advantages. First, NST has a rapid sequencing speed. A MinION flow cell contains 512 channels with 4 nanopores in each channel, a total of 2,048 nanopores used to sequence. The speed of bases passing through nanopores can reach up to 450 bp/s, and the yield per flow cell achieve approximately 10–15 GB in 24 h (Wang et al., 2021). Second, NST produces ultra-long reads. The average read length generated by a nanopore sequencer typically exceeds 500 bp, with a maximum read length up to 2.273 Mb (Payne et al., 2019). Also, some researchers propose that the read length of a nanopore sequencer depends on the length of the input DNA fragments. Jain et al. predicted through modeling that apart from the physical forces causing DNA breakage in the solution, nanopore sequencing might have no inherent limit on read length (Jain et al., 2018), enabling comprehensive analysis of the complex genome structure of microorganisms. Third, NST enables real-time sequencing. For instance, the MinION sequencing platform, a portable real-time sequencer, processes sequencing reads as they are generated, reducing the time for sequencing to just a few hours or even as little as 1 h (Taxt et al., 2020; Li et al., 2021), facilitating early and rapid clinical diagnoses. Fourth, NST allows single-molecule sequencing. Unlike NGS, nanopore sequencing does not rely on PCR-based signal amplification, enabling direct detection of DNA or RNA in samples at the single-molecule level and avoiding biases introduced by NGS in amplification and library preparation. Moreover, RNA virus genomes can be assembled directly, and RNA methylation can be detected simultaneously (Liu et al., 2019). Lastly, NST is convenient and cost-effective. For example, the MinION sequencer is portable, as small as a USB drive, costs approximately $1,000, and has a low per-run cost, making it highly suitable for clinical applications. The technological advantages of NST compared with NGS is shown in Table 1. These features and advantages have enabled the quiet integration of NST into clinical diagnosis, treatment, and scientific research.

**Table 1:** Technological advantages of NST compared with NGS.

Items	Nanopore sequencing technology (NST)	Next-generation sequencing (NGS)
Sequencing speed	10–15 GB/24 h/flow cell	10–15 GB/24 h
Read length	500 bp–2.273 Mb; depend on the length of the input DNA fragments	<500 bp
Sequencing turnaround time^a^	<6 h	>20 h
Sequencing method	Real-time single-molecule sequencing	Rely on PCR signal amplification
Practicability	The MinION, a portable sequencer, about $1,000	About $50,0000

a Refers to the cumulative time taken for nucleic acid extraction, reverse transcription, library preparation, sequencing, MetaPORE bioinformatics analysis, and pathogen detection.

## 4 Applying NST to diagnosis of BSI pathogens

### 4.1 Rapid detection of BSI pathogens

NST has great application value for the rapid diagnosis of a wide range of pathogens responsible for infections, including those affecting the respiratory tract (Mu et al., 2021; Bull et al., 2020), digestive system (Yonkus et al., 2022), urinary system (Zhang et al., 2022), central nervous system (Hong et al., 2020), intraocular area (Huang et al., 2021), and circulatory system (Liu et al., 2023). For instance, Hong et al. evaluated NST’s diagnostic performance of detection 202 blood samples from patients with hematological diseases, and 56 pathogens were identified in total, including bacteria, fungi, and viruses (Hong et al., 2023). The Sanger method was used to verify the accuracy of NST, and the results showed that compared with the combination of blood culture and PCR, which had clinical sensitivity and specificity of 66.67% and 40.11%, respectively, NST demonstrated substantially higher sensitivity and specificity of 92.11% and 78.41%, respectively. Additionally, the time from sample collection to final report for nanopore sequencing was under 24 h, with the preliminary reporting time for emergency cases reduced to <6 h. Therefore, NST holds promising potential for the rapid identification of broad-spectrum pathogens in BSI.

Furthermore, researchers have validated the accuracy and timeliness of NST in detecting various BSI pathogens. Ashikawa et al. (2018) used the MinION platform to sequence the 16S rRNA/ITS (Internal Transcribed Spacer) amplicons of 11 types of bacterial strains and 5 *Candida* species obtained from blood cultures, successfully identifying the pathogens within approximately 10 min of starting nanopore sequencing. Except for one strain each of *Streptococcus pyogenes* and *Escherichia coli*, the sequencing results from the first 10 min and after 48 h were consistent with those obtained using MALDI-TOF MS. The Sanger method was used to further validate MinION’s performance, showing average sequence identity rates of 99.03% in the first 10 min and 99.22% at 48 h for all 20 specimens. These results indicate that the reads obtained within the first 10 min of sequencing are sufficient for pathogen identification. This finding demonstrates that nanopore sequencing can rapidly and accurately detect common bacterial and fungal pathogens of BSI.

In addition to detecting common pathogens, nanopore sequencing demonstrates advantages in identifying virus and rare and fastidious bacteria. Greninger et al. (2015) utilized MinION nanopore sequencing coupled to a newly developed MatePORE bioinformatics pipeline for real-time bioinformatics analysis in four blood samples from virus-infected patients, achieving sample-to-detection time of under 6 h. At titers ranging from 10^7^–10^8^ copies/mL, reads to Ebola virus from two patients with acute hemorrhagic fever and chikungunya virus from an asymptomatic blood donor were detected within 4–10 min of data acquisition, while lower titer hepatitis C virus (1 × 10^5^ copies/mL) was detected within 40 min. Bialasiewicz et al. (2019) used nanopore sequencing to detect *Capnocytophaga canimorsus* in the whole blood of a patient bitten by a dog within just 19 h, whereas the patient’s blood culture remained negative even after 3 days. Wang et al. (2022) reported a case in which a patient undergoing allogeneic hematopoietic stem cell transplantation developed diarrhea and fever approximately 3 months after transplant. Gram-negative bacilli grew after 10 days of blood culture. The positive blood cultures were transferred to a blood agar plate for further culturing, but the culture failed, and MALDI-TOF MS detection was also unsuccessful. Researchers then extracted DNA from the positive blood cultures and performed nanopore sequencing, successfully identifying *Legionella pneumophila* subspecies *fraseri* within 1 h. The infection was ultimately controlled by adjusting the patient’s antibiotic regimen. These findings highlight the potential of nanopore sequencing in rapidly identifying viruses and rare pathogens, optimizing antibiotic use, and improving the prognosis of patients with BSI.

### 4.2 Detection of drug resistance and resistance genes in pathogens

Nanopore sequencing enables not only real-time sequencing but also the complete genome sequencing of pathogens, providing additional information such as drug resistance genes and aiding clinicians in selecting appropriate antibiotics and treatment plans. In a prospective, observational multicenter study conducted in Australia, 52 positive blood cultures from patients with clinical features of sepsis were sequenced using the MinION platform to evaluate the performance of ONT in identifying pathogens and predicting antimicrobial susceptibility (Harris et al., 2024). The results showed 27 types of gram-positive bacteria and 23 types of gram-negative bacteria were identified in the positive blood culture broths, with 2 samples containing polymicrobial infections. Compared with conventional methods, the species-level consistency of sequenced samples was 94.2% (49/52), and the accuracy for single-bacterial samples was as high as 98% (49/50). Among the 24 samples available for AST prediction based on high-quality sequencing, the overall agreement of the 262 AST results was 89.3%. These findings demonstrate that nanopore sequencing provides high accuracy in species identification and AST prediction.

Meanwhile, Liu et al. (2023) compared pathogen species identification and drug resistance gene detection using nanopore sequencing with traditional methods. The sequencing results of 37 positive strains isolated from blood cultures were consistent with those of MALDI-TOF MS. Due to the discharge or death of some patients, AST was performed on 32 of the 37 samples. Additionally, nanopore sequencing was conducted on 3 strains of *Staphylococcus aureus*, 10 strains of *E. coli*, and 7 strains of *Klebsiella pneumoniae* with standard AST phenotypes. Several antimicrobial resistance (AMR) genes corresponding to resistant phenotypes were detected across all three types of strains. For instance, *E. coli* and *K. pneumoniae* were found to carry *tet(A)*, which is associated with tetracycline resistance, and *dfrA*, which is associated with trimethoprim-sulfamethoxazole resistance. *S. aureus* carried *ermA*, which is associated with lincosamide and macrolide resistance. Additionally, *K. pneumoniae* harbored *blaKPC-2* (encoding serine carbapenemase) and *blaNDM-5* (encoding metallo-β-lactamase), which are associated with carbapenem resistance. However, some AMR genes, such as *ANT(9)-Ia*, which is associated with aminoglycoside resistance in *S. aureus*, showed no confirmed relationship with phenotypic AMR. Although the AMR gene profile does not always correlate with the resistance phenotype, accurately identifying these relationships can improve antimicrobial therapy outcomes, optimize personalized treatment regimens, and increase cure and survival rates in patients with BSI.

### 4.3 Analysis of the genomic characteristics and genetic background of pathogens

The repeated sequences and plasmids of microbial genomes often contain crucial genetic information related to drug resistance (De Maio et al., 2019). Nanopore sequencing, with its ultra-long reads, can not only assemble complete bacterial chromosome structures but also precisely identify complex multidrug resistance (MDR) gene islands on bacterial chromosomes and the structure of MDR plasmids carried by bacteria (Naushad et al., 2020). For example, Ashton et al. (2015) used long-read nanopore sequencing with the MinION platform, combined with short-read sequencing data for hybrid assembly, to successfully identify a complex drug resistance gene island inserted into the *Salmonella* chromosome, confirming its accuracy through experimental validation. Similarly, Li et al. (2023) isolated a strain of O139 *Vibrio cholerae* from the blood of a patient with bacteremia and obtained its complete genome sequence using nanopore sequencing. This strain contained 15 drug resistance genes, with two resistance islands carrying IS26 insertions capable of capturing and expressing foreign genes. Additionally, the strain harbored a 172,914-bp IncA/C plasmid, a drug-resistant plasmid with a strong ability to accumulate antibiotic resistance genes (Wang et al., 2015; Han et al., 2018), making the strain resistant to multiple antibiotics and enabling direct transfer of resistance genes through recombination with other plasmids. Furthermore, Tian et al. isolated a carbapenem-resistant *K. pneumoniae* strain from the blood of a patient with BSI complicated by liver abscess (Tian et al., 2022). Whole genome sequencing (WGS) performed using the Illumina and MinION platforms revealed that the strain contained one circular chromosome and nine plasmids. The carbapenemase-encoding gene *blaOXA-232* was located in a 6,141-bp ColKP3-type replicon flanked by ΔISEcp1 and ΔlysR–ΔereA, whereas *blaCTX-M-15* was located on the chromosome, mediated by the ISEcp1-based transposon Tn2012. Moreover, the strain carried an *rmpA2*-associated pLVPK-like virulence plasmid with one *iutA-iucABCD* gene cluster and an IS26-mediated MDR fusion plasmid. These findings contribute to a deeper understanding of the molecular mechanisms underlying drug resistance and the dynamic processes of resistance transmission. They are of significant importance for the prevention and control of drug resistance and the development of new antibiotics.

## 5 Discussion

Nanopore sequencing offers numerous advantages for the diagnosis of clinical infectious diseases. It can rapidly and precisely detect BSI pathogens, analyze resistance genes and genomic characteristics, and address the limitations of traditional etiological diagnostic methods and NGS, holding great potential for optimizing clinical BSI diagnostic procedures. Moreover, NST leverages the wealth of information from WGS data to assist in more precise determinations of molecular mechanisms underlying drug resistance and resistance transmission. This significantly contributes to the prevention and control of the spread of drug-resistant bacteria, as well as offers new ways and insights to develop antibiotics.

Additionally, nanotechnology and nanoparticles were developed in recent years to combat resistant bacteria and multidrug-resistant *Candida auris* (Hetta et al., 2023a, 2023b). Nanotechnology-based delivery systems are increasingly being evaluated as viable options for improving therapeutic efficacy by limiting drug degradation, increasing accumulation at infection sites, and reducing toxicity. While nanoparticles, as transporters for natural antibiotics and antimicrobials, are not only able to carry medications to the target location in the ideal dosage range, but also actively combat bacteria. Therefore, nanoparticles are considered a targeted therapy approach.

However, NST still has its own limitations. First, the error rate associated with nanopore sequencing can reach 5–15% (Rang et al., 2018), significantly higher than that of NGS at 0.1–1%. The high error rate represents a major drawback of NST, it may potentially result in misidentification of pathogens with similar genomic sequences (Sakai et al., 2019). Therefore, researchers have been working to employ 2D and 1D^2^ library preparation methods to enable repeated sequencing of DNA fragments (Rhoads and Au, 2015), enhancing the accuracy of nanopore sequencing. Second, nanopore sequencing is susceptible to interference from the host genome or other background elements, imposing stringent requirements on sample quality and nucleic acid abundance. To improve detection sensitivity, pretreatment such as purification or targeted enrichment of microbial nucleic acids are necessary. Lastly, although the per-run cost of NST is relatively low, its sequencing throughput is lower than that of NGS (Laver et al., 2015). Consequently, obtaining the same amount of data results in increased operational costs for nanopore sequencing, thus limiting its broad application.

## 6 Conclusion

NST has been applied in studies involving animals, plants, and microbial samples. Its clinical applications primarily focus on genetics, immunology, oncology and microbiology. As a TGS technology, NST poses promising prospects and significant potential application values. However, NST remains considerable room for enhancement in both product performance and clinical application effect. Particularly, the high error rate in single nucleotide detection and the requirement for high initial nucleic acid concentrations during library preparation pose substantial limitations on the further application of NST in advantageous areas. Currently, integrating the advantages of NST and NGS could more effectively address the challenges encountered in both clinical practice and scientific research.
